# Accuracy of intraocular lens calculation formulas in cataract patients with steep corneal curvature

**DOI:** 10.1371/journal.pone.0241630

**Published:** 2020-11-20

**Authors:** Chenguang Zhang, Guangzheng Dai, Emmanuel Eric Pazo, Ling Xu, Xianwei Wu, Hongda Zhang, Tiezhu Lin, Wei He

**Affiliations:** 1 Department of Ophthalmology, He Eye Specialists Hospitals, Shenyang, China; 2 Eye Hospital and School of Ophthalmology and Optometry, Wenzhou Medical University, Wenzhou, Zhejiang, China; University of Toronto, CANADA

## Abstract

**Objective:**

To compare the accuracy of five kinds of intraocular lens calculation formulas (SRK/T, Haigis, Hoffer Q, Holladay and Barrett Universal Ⅱ) in cataract patients with steep curvature cornea ≥ 46.0 diopters.

**Methods:**

This is a retrospective study of cataract phacoemulsification combined with intraocular lens implantation in patients with steep curvature cornea (corneal curvature ≥ 46D). The refractive prediction errors of IOL power calculation formulas (SRK/T, Haigis, Holladay, Hoffer Q, and Barrett Universal II) using User Group for Laser Interference Biometry (ULIB) constants were evaluated and compared. Objective refraction results were assessed at one month postoperatively. According to axial length (AL), all patients were divided into three groups: short AL group (<22mm), normal AL group (>22 to ≤24.5mm) and long AL group (>24.5mm). Calculate the refractive error and absolute refractive error (AE) between the actual postoperative refractive power and the predicted postoperative refractive power. The covariance analysis was used for the comparison of five formulas in each group. The correlation between the absolute refractive error and AL from every formula were analyzed by Pearson correlation test, respectively.

**Result:**

Total 112 eyes of 83 cataract patients with steep curvature cornea were collected. The anterior chamber depth (ACD) was a covariate in the short AL group in the covariance analysis of absolute refractive error (*P*<0.001). The SRK/T and Holladay formula had the lowest mean absolute error (MAE) (0.47D), there were statistically significant differences in MAE between the five formulas for short AL group (*P* = 0.024). The anterior chamber depth had no significant correlation in the five calculation formulas in the normal AL group and long AL group (*P* = 0.521, *P* = 0.609 respectively). In the normal AL group, there was no significant difference in MAE between the five calculation formulas (*P* = 0.609). In the long AL group, Barrett Universal II formula had the lowest MAE (0.35), and there were statistically significant differences in MAE between the five formulas (*P* = 0.012). Over the entire AL range, the Barrett Universal II formula had the lowest MAE and the highest percentage of eyes within ± 0.50 D, ± 1.00 D, and ± 1.50 D (69.6%, 93.8%, and 98.2% respectively).

**Conclusion:**

Compared to SRK/T, Haigis, Hoffer Q, and Holladay, Barrett Universal Ⅱ formula is more accurate in predicting the IOL power in the cataract patients with steep curvature cornea ≥ 46.0 diopters.

## Introduction

Cataract surgery is one of the most commonly performed procedures in the increasingly ageing population. Postoperative refractive error is one of the primary cause of postoperative visual outcome dissatisfaction among patients. Thus, accurate IOL power calculations have become extremely important.

The calculation formula of artificial crystal has gradually developed from the first generation of theoretical formula to the Hill-radial basis function formula [1]. Yet there is still considerable debate about which formula provides the most accurate refractive prediction. Some scholars found that Hoffer Q performed best for eyes shorter than 22.0 mm [2], while most modern theoretical IOL formulas perform well for eyes with the normal eye axis lengths (22.0–24.5 mm) [3], Barrett Universal Ⅱ formula is considered to be most accurate in the long axis lengths [4, 5].

Borish and Duke-Elder classification of myopia as an optical system suggests 3 forms of myopia. Myopia due to increased axial length of the eye (axial myopia), refractive myopia and curvature myopia [6]. Although, AL is considered to be most important parameter in most modern-day formulae as it can changes the IOL power by nearly 2.5 to 3 times, more so in short eyes than in longer ones [7]. K readings are considered the second most important factor necessary for accurate IOL power calculations. Changes in K reading can alter the IOL power in a ratio of nearly 1:1 ratio [8]. As for whether the corneal curvature variation will affect the measurement of intraocular lens power, especially in the case of large corneal curvature, there is a paucity of research in their area. Therefore, this study assessed cataract patients with steep corneal curvature (corneal curvature≥ 46.0D). Five formulas were used to calculate the refractive power of the IOLs, namely the SRK / T [9], Haigis [10], Hoffer Q [11], Holladay [12], and Barrett Universal Ⅱ [13] formulas. Although, the accuracy of IOL power calculation formulas for highly myopic eyes have been assessed by Zhang et al. [5] on patients’ keratometric value ranging from 40.36 to 48.19 D (mean: 43.61±2.22 D), our current study includes a higher range of keratometric values (range: 46.00 to 51.03 D; mean: 47.31±1.08 D). Therefore, the purpose of this study is to assess the predictive accuracy of various IOL power formulas in eyes with steep corneal curvature.

## Patients and methods

The study was approved by the Ethics Committee of the He Eye Specialists Hospitals, Shenyang on January 2020 and all research was performed in accordance with the Declaration of Helsinki.

This was a retrospective study of cataract phacoemulsification combined with intraocular lens implantation at He Eye Specialists Hospitals, Shenyang included patient data from January to June 2019 with steep corneal curvature (corneal curvature ≥ 46D) from the central hospital database.

Cataract extraction with IOL implantation was performed at He Eye Specialists Hospitals, Shenyang China by the practical senior surgeons. Cases from January 2019 to June 2019, were reviewed.

The inclusion criteria were as follows: (1) biometric measurements determined by PCI (IOLMaster, Software V5.4 and above, Carl Zeiss Meditec, Inc., Dublin, CA, USA); (2) cataract surgery performed by phacoemulsification and in-the-bag IOL implantation; (3) use of the Hoya IOL (Hoya-PS AF-1 Series, Model PC-60AD, Hoya, Japan); and (4) 2.75 mm clear corneal incisions located temporally or superiorly. The exclusion criteria were as follows: (1) patients with a history of previous intraocular surgery or intraoperative or postoperative complications; (2) preexisting ocular diseases that may influence postoperative refraction, including keratoconus, corneal scarring, endothelial dystrophy, retinal detachment, and macular edema; (3) patients undergone prior refractive surgical procedures including refractive laser correction; (4) patients with follow-up of less than 1 month. In order to accurately measure all parameters required for IOL power calculation (SRN>2.0, including AL, anterior chamber depth (ACD), and keratometric (K) (both K 1 and K 2) values were collected for the back calculation of formulas. We selected cataract patients with corneal curvature ≥46D, without pterygium or keratoconus. Patients with intraoperative complications such as posterior capsule rupture, lens nucleus dislocation into the vitreous cavity, sulcus, or sutured lens were also excluded from the analysis. All data included for the final analysis was anonymized and coded in serial numbers. The authors of this current study were not allowed access to information that could identify individual participants during or after data collection.

Eyes were divided into three groups according to their AL. The three groups consisted of short axis group (AL≤22mm), normal axis group (22mm <AL≤24.5mm) and long axis group (AL> 24.5mm).

The IOL calculation results of SRK/T, Haigis, Hoffer Q, and Holladay formulas were obtained from IOL Master 500 and the calculation results of the Barrett Universal Ⅱ formula were obtained online. The objective post-operative refraction was assessed 1 month postoperatively. The prediction error is defined as the actual postoperative spherical equivalent (SE) that was calculated by each IOL formula minus the predicted postoperative SE. Thus, a positive predicted error in refraction indicates a hyperopic result than the predicted refraction. The absolute refractive error (AE) also were calculated as well as the percentage of eyes that had a predicted refractive error of within ±0.50 D, ± 0.75 D, and ±1.00 D. we assessed correlations between AL and AE.

Statistical comparisons between formula absolute errors were performed using repeated measures analysis of variance (ANOVA). The relationship between the AE and AL was analyzed by Pearson correlation test. Statistical significance was defined as *P*< 0.05.

## Results

The final analysis consisted of 112 eyes from 83 patients with steep corneal curvature (corneal curvature ≥46D) who underwent cataract surgery. Of the 83 patients, 64 (77.11%) were female, and the ages ranged from 45 to 91 years (mean = 70.04 ± 9.75 years). The ALs of the study population ranged from 19.83 to 31.92 mm (mean = 23.30±2.37 mm), the preoperative corneal curvatures from 46.00 to 51.03 D (mean = 47.31±1.08 D), and the preoperative anterior depths from 2.06 to 4.11 mm (mean = 2.97±0.44 mm) (Table 1).

**Table 1:** Study population characteristics.

Parameter	Mean	SD	Min	Max
Age (year)	70.04	9.75	45	91
AL (mm)	23.30	2.37	19.83	31.92
K (D)	47.31	1.08	46.00	51.03
ACD (mm)	2.97	0.44	2.06	4.11

AL = axial length; K = keratometry; ACD = anterior chamber depth.

Table 2 shows the results of five formulas for different AL groups. The MAE varied with respect to AL with all 5 formulas. The anterior chamber depth was a covariate in the short axis eye group in the covariance analysis of absolute refractive error (p <0.001), and had no significant correlation in the five calculation formulas in the normal AL group and long AL group (P = 0.521, P = 0.609 respectively). There were statistically significant differences in MAE between the 5 formulas for the short AL group and long AL group (P = 0.024, P = 0.012 respectively). The Haigis formula has the highest MAE (0.77D). The SRK/T and Holladay formula has the lowest MAE (0.47D) and is close to the Barrett formula (0.48D). In the normal AL group, the ANOVA showed the difference between the 5 formulas was not statistically significant (P = 0.608). In the normal AL group and long AL group, the lowest MAE was achieved with the Barrett Universal II formula (0.41D, 0.35D respectively). In the whole sample, the lowest MAE was achieved once more with the Barrett Universal II formula.

**Table 2:** Mean absolute predicted error by five formulas.

Formula	MAE (D)			
	AL≤22mm (n = 41)	22<AL≤24.5mm (n = 45)	AL>24.5mm (n = 26)	entire AL(n = 112)
SRK/T	0.47	0.46	0.78	0.54
Haigis	0.77	0.51	0.51	0.61
Hoffer Q	0.51	0.45	0.76	0.54
Holladay	0.47	0.41	0.68	0.49
Barrett	0.48	0.41	0.35	0.42
P value	0.024*	0.608	0.012*	0.037*

AL = axial length; MAE = Mean absolute error; Barrett = Barrett Universal II; *indicates P<0.05.

Table 3 shows in the short eye axis group, the mean refractive errors of the five formulas are all positive. In the SRK/T formula, the mean refractive error of each group is also positive.

**Table 3:** Mean predicted error by five formulas.

Formula	ME (D)			
	AL≤22mm (n = 41)	22<AL≤24.5mm (n = 45)	AL>24.5mm (n = 26)	entire AL(n = 112)
SRK/T	-0.26	-0.28	-0.29	-0.28
Haigis	-0.65	-0.29	0.27	-0.29
Hoffer Q	-0.13	0.25	0.72	0.22
Holladay	-0.01	0.11	0.30	0.11
Barrett	-0.08	0.02	0.11	0.003
P value	<0.001*	<0.001*	<0.001*	<0.001*

AL = axial length; MAE = Mean predicted error; Barrett = Barrett Universal II; *indicates P<0.05.

Table 4 shows the percentage of eyes whose predicted refractive outcome for each formula are within ± 0.50 D, ± 0.75 D, or ± 1.00 D. In short AL group, the Hoffer Q formula had the highest percentage of eyes within ± 0.50 D, ± 1.00 D, and ± 1.50 D (70.0%, 90.2%, and 97.6% respectively). In long AL group and the entire AL range, the Barrett Universal II formula had the highest percentage of eyes within ± 0.50 D, ± 1.00 D, and ± 1.50 D. In the normal axis group, the refractive error of most eyes within ± 1.00 D.

**Table 4:** Percentage of eyes within predicted error ranges.

Formula	AL≤22mm(%)			22<AL≤24.5mm(%)			AL>24.5mm(%)			entire AL(%)		
	≤±0.5D	≤±1.0D	≤±1.5D	≤±0.5D	≤±1.0D	≤±1.5D	≤±0.5D	≤±1.0D	≤±1.5D	≤±0.5D	≤±1.0D	≤±1.5D
SRK/T	65.9	90.2	97.6	66.7	91.1	95.6	46.2	73.1	80.8	61.6	86.6	92.9
Haigis	34.1	65.9	92.7	48.9	91.1	100	53.9	92.3	96.2	44.6	82.1	96.4
Hoffer Q	70.0	90.2	97.6	64.4	88.9	100	38.5	69.2	88.5	57.1	84.8	96.4
Holladay	68.3	90.2	97.6	66.7	97.8	100	46.2	76.9	92.3	62.5	90.2	97.3
Barrett	63.4	90.2	95.1	71.1	95.6	100	76.9	96.2	100	69.6	93.8	98.2

AL = axial length; Barrett = Barrett Universal II.

**Fig 1:**
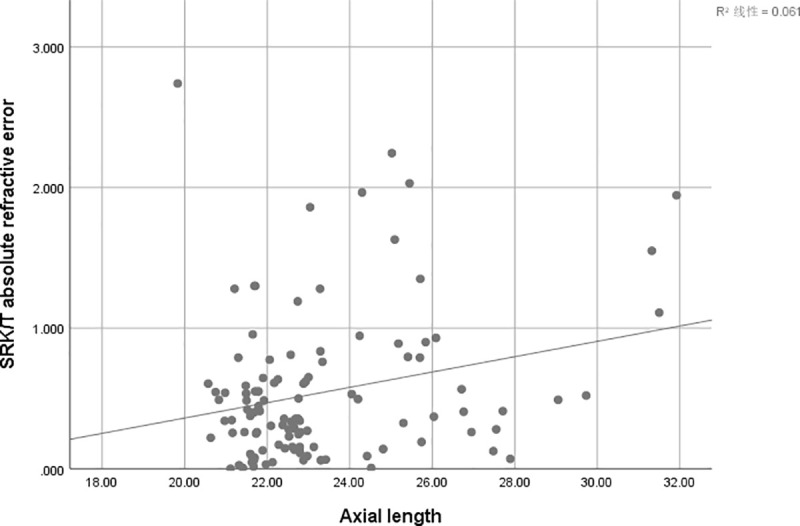
Correlations between axial length and absolute error. The associations between axial length and absolute error were analyzed using absolute errors derived from SRK/T formula (r = 0.247, r^2^ = 0.061; P = 0 .009).

**Fig 2:**
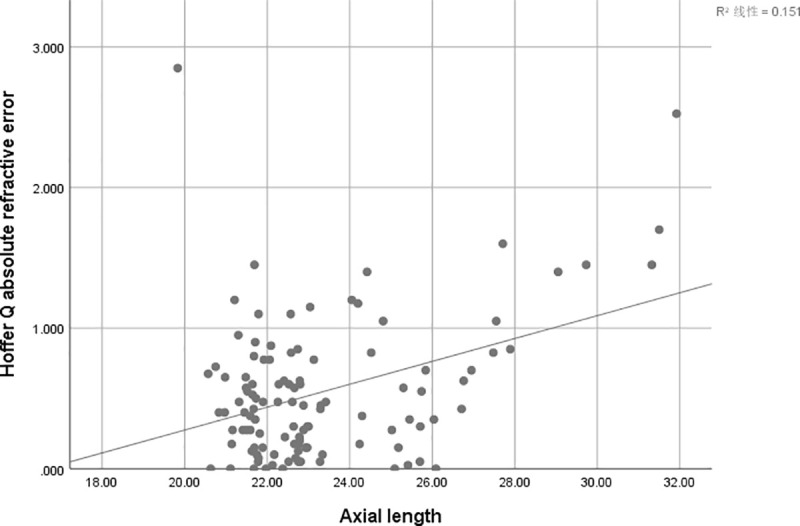
Correlations between axial length and absolute error. The associations between axial length and absolute error were analyzed using absolute errors derived from Hoffer Q formula (r = 0.388, r^2^ = 0.151; P<0.001).

**Fig 3:**
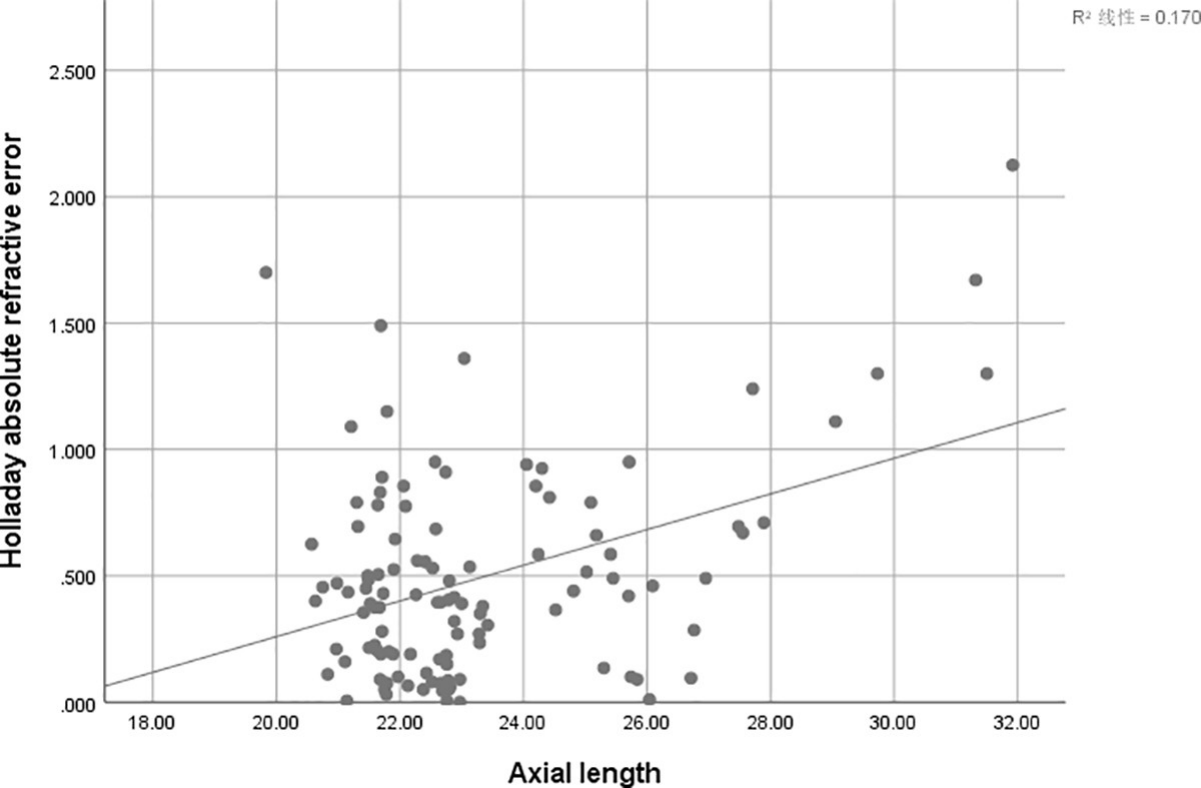
Correlations between axial length and absolute error. The associations between axial length and absolute error were analyzed using absolute errors derived from Holladay formula (r = 0.412, r^2^ = 0.170; P<0.001).

**Fig 4:**
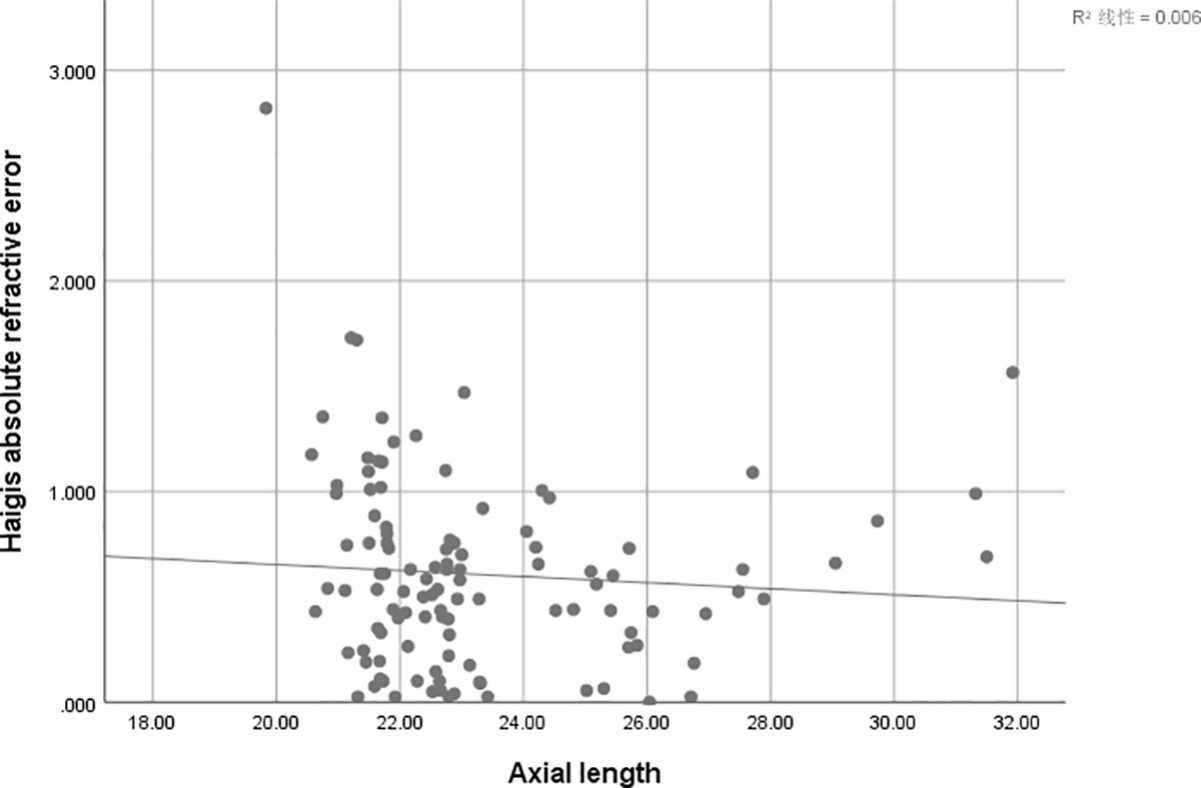
Correlations between axial length and absolute error. The associations between axial length and absolute error were analyzed using absolute errors derived from Haggis formula (r = -0.076, r^2^ = 0.006; P = 0.428).

**Fig 5:**
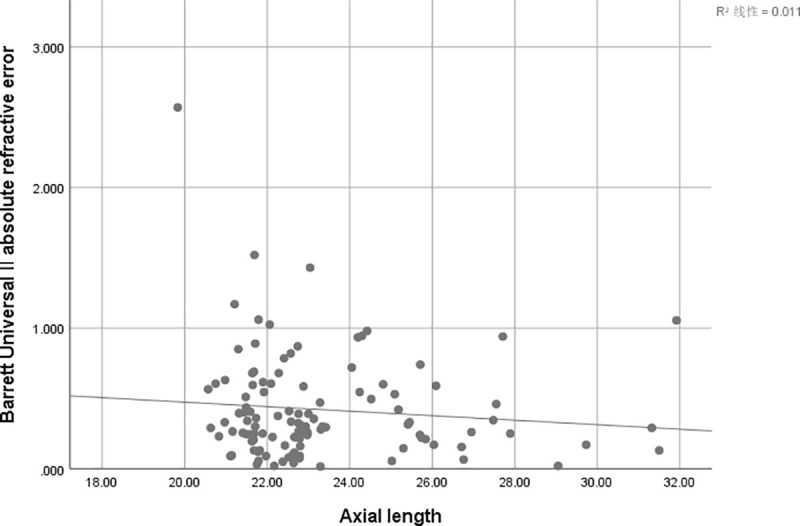
Correlations between axial length and absolute error. The associations between axial length and absolute error were analyzed using absolute errors derived from Barrett Universal II formula (r = -0.103, r^2^ = 0.011; P = 0.278).

There is a positive correlation between AE and AL of SRK/T (r = 0.247, r^2^ = 0.061; P = 0 .009) (Fig 1), Hoffer Q (r = 0.388, r^2^ = 0.151,P<0.001) (Fig 2)and Holladay (r = 0.412, r^2^ = 0.170; P<0.001) (Fig 3), but not for Haigis (P = 0.428) (Fig 4), and Barrett Universal II formula (P = 0.278) (Fig 5).

## Discussion

Althought, the accuracy of IOL power calculation formulas for highly myopic eyes have been assessed by Zhang et al. [5] (mean K: 43.61±2.22 D), our current study includes a higher range of keratometric values (range: 46.00 to 51.03 D; mean K: 47.31±1.08 D). Our study did not include keratoconic eyes but we had eyes with high corneal curvature, in general IOL power calculation formulas are assumed for normal eyes. Studies such as Watson et al. [14] have reported that while hyperopic error was low in eyes with mild (K < 48.00 D) and moderate (K = 48.00–55.00 D) corneal curvature eyes following cataract surgery, However, errors can be unpredictable in severe steepening of the cornea. Studies have found that in normal eyes, the refractive outcomes predicted by modern formulas are within ± 0.5 D of the actual postoperative refractive outcomes in about 75% of eyes with general curvature cornea [15–17]. However, in this study, all the eyes are with steep curvature cornea, most formulas only achieved a ±0.5 D prediction accuracy in 70% of eyes with the short AL group and normal AL group. In the long AL group, the Barrett Universal II formula achieved a ±0.5 D predictive accuracy in 76.9% of eyes, with most other formulas only achieving around 50% accuracy. Over the entire AL range, the Barrett Universal II was the most accurate formula by a significant margin, had a highest percentage of eyes with prediction errors between ±0.5 D, ±1.0 D, and ±1.50 D than the other 4 formulas assessed. Cooke et al. [15] found Barrett Universal II had the highest percentage of eyes with prediction errors between± 0.5 D and ±1.0 D than other formulas in all eyes. In this study, the percentage of eyes within ±0.5 D in most formulas is much lower than the reported in normal eyes, probably because the relationship between corneal curvature, ACD and IOL position changes, thus reducing the accuracy of formula in predicting the effective lens position.

In short AL group, the average refractive errors of the five formulas are negative, the mean refractive error of the Haigis formula was the highest (-0.65), and the Holladay formula was the lowest (-0.01D), which is close to the Barrett Universal II formula (-0.08D). All 5 formulas assessed in this study resulted in mean myopic predicted errors. The same conclusion can be found in the study by Kane et al. [17] For patients in short AL, the choice of intraocular lens power can be appropriately small. In long AL group, except for the SRK / T formula, the mean refractive error of the other four formulas are all positive, indicates that such patients have hyperopic predicted errors. Chen et al. [18] found that most eyes with an AL of >33 mm presented with postoperative hyperopia of +2.0 D to +3.0 D. For patients with long ALs, especially with extremely long ALs, the reserved intraocular lens power should be increased.

Gavin et al. [2] analyzed the British short eyes patients and found that the Hoffer Q formula is more accurate. Aristodemou et al. [19] showed that HofferQ performed the best on AL from 20.00 to 20.99 mm, and Hoffer Q and Holladay 1 performed best on AL from 21.00 to 21.49 mm. Day et al. [20] reported that in patients with AL ≤ 22mm, the Hoffer Q, Holladay, and Haigis formulas have best accuracy, and the SRK / T formula has a large error. In our study, in short group, the Holladay and SRK/T formulas having the lowest MAE followed by Barrett Universal II and Hoffer Q formulas. The Haigis formulas had the highest MAE. There were statistically significant differences in MAE between the 5 formulas (P = 0.024). Similar to Kane et al. [17] we also found that short ALs, the Holladay 1 formula had the lowest MAE followed by SRK/T, Holladay 2, and Barrett Universal II formulas, while Haigis and Hoffer Q formulas had the higher MAE.

In the normal axis group, the lowest MAE was achieved with the Barrett Universal II and Holladay formulas (0.41), but the difference between formulas was not statistically significant. The most accurate IOL power formula in the normal AL group was not clearly defined. Most researchers believe that the accuracy of various formulas is similar. Reitblat et al. [3] study on Holladay 1, SRK/T, Hoffer Q, Haigis, Holladay 2, Barrett Universal II and Olsen formulas in this AL range did not find any formula to be more accurate.

In our study, we found that the most accurate formula was Barrett Universal II followed by SRK/T, Haigis, Holladay and Hoffer Q in long AL group. There were statistically significant differences in MAE between the 5 formulas (P = 0.012). Zhang Y et al. [5] found through comparison and analysis of patients with AL of 26.00 mm or above, Barrett Universal II formula has the lowest absolute refractive error compared to Haigis, Hoffer Q, Holladay, and SRK / T formulas.

Over the entire AL range, Barrett Universal II formula has the lowest absolute refractive error and was the most accurate formula than the other 4 formulas. Melles et al. [16] found that the MAE of Barrett Universal II formula in each eye axis group is the lower. Barrett Universal II formula is still more accurately with steep corneal curvatures. Kendrick et al. [21] found that Barrett Universal II formula was superior in eyes with stage I (corneal powers of 48 D or lower) and stage II (corneal power between 48.01 D and 53 D) keratoconus. The results are the same as our research.

Our study has several limitations. First, this study is retrospective, and a randomized controlled study may provide more detailed clinical information. Second, the sample size was small, especially for long axis. However, this is the first paper to present the IOLs calculation in the eyes with steep curvature corneal curvature ≥ 46.0 diopters. Additionally, modern formulas such as Holladay 2 and Oslen were not available at our disposal and therefore we were unable to assess their performance. Since the Olsen is a ray-tracing thick-lens formula and includes variables such as the AL, K value, ACD, lens thickness, and age of the patient for calculation [22], it theoretically can perform at par with that Barrett Universal Ⅱ formula [4].

In conclusion, Barrett Universal Ⅱ formula is more accurate than other calculation formulas in predicting the IOL degrees in cataract patients with steep curvature cornea, and the impact of AL is less compared to others. Further research is warranted using modern IOL formulas in order to understand the impact of steep curvature cornea on post-surgical refractive outcomes in cataract and clear lens extraction patients.

## References

[1] Roberts TV, Hodge C, Sutton G, et al Comparison of Hill‐radial basis function, Barrett Universal and current third generation formulas for the calculation of intraocular lens power during cataract surgery[J]. Clinical & Experimental Ophthalmology, 2018, 46(3):240–246. 28778114 10.1111/ceo.13034

[2] Gavin EA, Hammond CJ. Intraocular lens power calculation in short eyes[J]. Eye, 2008, 22(7):935–938. 10.1038/sj.eye.6702774 17363925

[3] Reitblat O, Assia E I, Kleinmann G, et al Accuracy of predicted refraction with multifocal intraocular lenses using two biometry measurement devices and multiple intraocular lens power calculation formulas[J]. Clinical & Experimental Ophthalmology, 2015, 43(4):328–334. 25491591 10.1111/ceo.12478

[4] Rong Xianfang et al Intraocular lens power calculation in eyes with extreme myopia: Comparison of Barrett Universal II, Haigis, and Olsen formulas[J]. J Cataract Refract Surg 2019; 45:732–737. 10.1016/j.jcrs.2018.12.025 30876784

[5] Zhang Y, Ying Liang X, Liu S, Lee JWY, Bhaskar S, Lam DSC. Accuracy of intraocular lens power calculation formulas for highly myopic eyes[J]. Journal of Ophthalmology, 2016(2016):1–7. 10.1155/2016/1917268 PMC4828549 27119018

[6] Duke-Elder Sir Stewart (1969). The Practice of Refraction (8th ed)[M]. St. Louis: The C.V. Mosby Company. ISBN 0-7000-1410-1.

[7] Bhardwaj V, Rajeshbhai GP. Axial Length, Anterior Chamber Depth-A Study in Different Age Groups and Refractive Errors[J]. Journal of Clinical And Diagnostic Research. 2013;7(10):2211–12. 10.7860/JCDR/2013/7015.3473 24298478 PMC3843406

[8] Mingue K, Youngsub E, Hwa L, et al Use of the Posterior/Anterior Corneal Curvature Radii Ratio to Improve the Accuracy of Intraocular Lens Power Calculation: Eom's Adjustment Method[J]. Investigative ophthalmology & visual ence, 2018, 59(2):1016. 10.1167/iovs.17-22405 29450545

[9] Retzlaff J A, Sanders D R, Kraff M C. Development of the SRK/T intraocular lens implant power calculation formula[J]. Journal of Cataract & Refractive Surgery, 1990, 16(3):333–340. 2355321 10.1016/s0886-3350(13)80705-5

[10] Haigis W, Lege B, Miller N, et al Comparison of immersion ultrasound biometry and partial coherence interferometry for intraocular lens calculation according to Haigis[J]. Graefe's Archive for Clinical and Experimental Ophthalmology, 2000, 238(9):765–773. 10.1007/s004170000188 11045345

[11] Hoffer Kenneth J. The Hoffer Q formula: A comparison of theoretic and regression formulas[J]. Journal of Cataract & Refractive Surgery, 1993, 19(6):700–712; Erratam in: Journal of Cataract and Refractive Surgery, 20: 677, 1994, and 33: 2–3, 2007. 10.1016/s0886-3350(13)80338-0 8271165

[12] Holladay JT, Musgrove KH, Prager TC, et al A three-part system for refining intraocular lens power calculations[J]. Journal of Cataract & Refractive Surgery, 1988, 14(1):17–24. 3339543 10.1016/s0886-3350(88)80059-2

[13] Barrett Graham D. An improved universal theoretical formula for intraocular lens power prediction[J]. Journal of Cataract & Refractive Surgery, 1993, 19(6):713–720. 8271166 10.1016/s0886-3350(13)80339-2

[14] Watson MP, Anand S, Bhogal M, et al Cataract surgery outcome in eye with keratoconus[J]. Br J Ophthalmol. 2014, 98(3):361–364. 10.1136/bjophthalmol-2013-303829 23966369

[15] Cooke DL, Cooke TL. Comparison of 9 intraocular lens power calculation formulas[J]. Journal of Cataract & Refractive Surgery, 2016, 42(8):1157–1164. 27531292 10.1016/j.jcrs.2016.06.029

[16] Melles RB, Holladay JT, Chang WJ. Accuracy of intraocular lens calculation formulas[J]. Ophthalmology, 2018, 125(2):169–178. 10.1016/j.ophtha.2017.08.027 28951074

[17] Kane JX, Van Heerden A, Atik A, et al Intraocular lens power formula accuracy: Comparison of 7 formulas[J]. Journal of Cataract & Refractive Surgery, 2016, 42(10):1490–1500. 27839605 10.1016/j.jcrs.2016.07.021

[18] Chen C, Xu X, Miao YY, et al Accuracy of intraocular lens power formulas involving 148 eyes with long axial lengths: A retrospective chartreview study[J]. J Ophthalmol, 2015(2015):1–7. 10.1155/2015/976847 PMC4697084 26793392

[19] Aristodemou P, Knox Cartwright NE, Sparrow JM, et al Formula choice: Hoffer Q, Holladay 1, or SRK/T and refractive outcomes in 8108 eyes after cataract surgery with biometry by partial coherence interferometry[J]. J Cataract Refract Surg, 2011;37(1): 63–71. 10.1016/j.jcrs.2010.07.032 21183100

[20] Day AC, Foster PJ, Stevens JD. Accuracy of intraocular lens power calculations in eyes with axial length <22.00 mm[J]. Clin Experiment Ophthalmol, 2012, 40(9): 855–862. 10.1111/j.1442-9071.2012.02810.x 22594574

[21] Kendrick M W, Albert S J, John G, et al Accuracy of Intraocular Lens Formulas in Eyes With Keratoconus[J]. Am J Ophthalmol, 2020, 4(212):26–33. 10.1016/j.ajo.2019.11.019 31770511

[22] Olsen T. Prediction of the effective postoperative (intraocular lens) anterior chamber depth[J]. Journal of Cataract & Refractive Surgery, 2006, 32(3):419–424. 16631049 10.1016/j.jcrs.2005.12.139

